# Implementation and utility of an online psychological assessment tool in youth soccer players: a one-year longitudinal study

**DOI:** 10.3389/fspor.2026.1733902

**Published:** 2026-01-26

**Authors:** Takashi Sano, Yuki Hamano

**Affiliations:** 1 Department of Sports Education, Chukyo University, Aichi, Japan; 2 Department of Research and Development, Trois-re Co., Ltd., Osaka, Japan

**Keywords:** digital tool, motivation, psychological state, satisfaction, soccer, youth athletes

## Abstract

Most studies on digital tools for assessing psychological states in sports have focused on screening athletes who show pathological tendencies, while few have examined their potential use for team management or coaching strategies. Moreover, little evidence exists regarding long-term, voluntary use among youth athletes. This study introduced an online psychological assessment system into a youth sports team to examine the effectiveness of digital tools for monitoring athletes’ psychological states, based on usage patterns and assessment results. Participants were 81 male soccer academy athletes aged 12–17 years. Analyses were conducted on annual participation frequency, overall score trends, intraindividual variability, and the relationships between motivation to continue participation and satisfaction measures. The athletes completed the assessments an average of 4.16 times over the year, but participation rates declined in the latter half of the period. The variance in relationship with coach and satisfaction with growth increased during the competitive season. Satisfaction with life and satisfaction with time in daily life showed large intraindividual fluctuations. Satisfaction with growth, relationship with coach, satisfaction with life, and satisfaction with time in daily life significantly influenced motivation to continue participation. These results indicate that psychological assessment tools may be useful for tracking seasonal changes in athletes’ psychological states, enabling early detection of dissatisfaction or concerns beyond team activities, and guiding the prioritization of support to promote sustained sport participation. Digital tools enabling regular psychological monitoring can reduce respondent burden, complement coaches’ expertise, and serve as effective resources for team management and mental health support.

## 1 Introduction

Monitoring athletes’ psychological states is an essential procedure for providing support to enhance performance and sustain participation. Pressures inherent to sport, competition among peers, and the occurrence of injuries can pose significant obstacles to continued involvement (1). Therefore, coaches must strive not to overlook the causes of athletes’ concerns or minor triggers that may affect their well-being. Because accurate assessment of psychological states requires specialized knowledge and skills, it is important to explore strategies that complement coaches’ expertise and enable precise understanding of athletes’ mental conditions.

As tools to supplement coach observation, digital systems focusing on athletes’ conditioning and mental health care have been developed, and their effectiveness and challenges have been reported (2, 3). Previously reported digital tools can be broadly categorized into training tools for psychological skill development and measurement tools for assessing psychological states. Training tools typically have clearly defined goals for improving psychological skills, allowing structured intervention periods and feasibility testing in sports teams (4–6). In contrast, measurement tools are often used to screen a subset of athletes showing pathological tendencies, for example using systems such as the SMHAT-1 (7–9), but there are few studies examining how measurement results can be applied to broader mental health care or inform team management and coaching strategies. Understanding usage patterns and the potential application of measurement results in team settings provides valuable information for improving athletes’ mental health and the activity environment.

Moreover, most studies on digital tools for psychological assessment have focused on university-level or older athletes (10), with limited research on youth athletes. In youth athletes, relationships with coaches, family, and teammates have been shown to influence motivation to continue participation (11). Adolescents also tend to engage in less self-disclosure than adults, with males particularly demonstrating lower offline self-disclosure (12). These characteristics suggest that youth athletes may find it difficult to express concerns directly, highlighting the potential value of digital tools for supporting these individuals.

The purpose of this study was to examine the effectiveness of a digital tool for monitoring psychological states by introducing an online psychological assessment system into a youth sports team and evaluating both usage patterns and the potential application of assessment results. Specifically, the study focused on examining usage patterns throughout the year, overall trends and variability in psychological scores, and the usefulness of assessment results as factors related to motivation to continue participation in team activities.

## 2 Method

### 2.1 Participants

The participants were 81 male youth athletes enrolled in a soccer academy operated by a professional Japanese soccer league (J-League) team. Their ages ranged from 12 to 17 years. The number of athletes by grade was as follows: 23 grade 7 athletes, 21 grade 8 athletes, 12 Grade 9 athletes, 14 Grade 10 athletes, and 11 Grade 11 athletes.

### 2.2 Overview of the online psychological assessment

The online psychological assessment used in this study was provided by Trois-re Co., Ltd. (NOCC for Sports). This system includes questionnaire items designed to measure respondents’ psychological states and has been utilized across sports teams in various disciplines to capture athletes’ characteristics and monitor their mental conditions. In particular, it has been introduced in youth teams, with reported cases including junior high school baseball clubs and lacrosse teams (13). The assessment procedure is as follows (see Supplementary Figure S1 for details):

1. Access the assessment QR code via a computer, smartphone, or tablet.
2. Enter the required information, check the consent screen, and start the assessment.
3. Respond to the items presented in a questionnaire format.
4. Upon completion, the results are immediately provided as feedback to the respondent.
5. Coaches with administrator privileges can view all athletes’ results within the team.

Table 1 lists the measures and questionnaire items included in the psychological assessment tool. The assessment includes eight measures assessing youth athletes’ motivation to continue participation, interpersonal relationships, and overall life satisfaction: motivation to continue participation (1 item), relationship with family (7 items), relationship with teammates (7 items), relationship with coach (4 items), satisfaction with home environment (1 item), satisfaction with life (5 items), satisfaction with time in daily life (1 item), and satisfaction with growth (1 item). All items were rated on a 5-point Likert scale (1 = strongly disagree, 2 = disagree, 3 = neither agree nor disagree, 4 = agree somewhat, 5 = strongly agree). When a measure included multiple items, the mean score of the items was used as the overall measure score.

**Table 1:** Measures and questionnaire items in the psychological assessment tool.

Measure	Item
1. Motivation to continue participation	• I want to continue participating in this team's activities.
2. Relationship with family	• My family does what I want them to do. • I am generally satisfied with my relationship with my family. • I think my relationship with my family is better than most peoples. • I sometimes wish I did not have a relationship with my family. (Reverse-coded) • My family meets my expectations. • I like my family. • There are problems between me and my family. (Reverse-coded)
3. Relationship with teammates	• My teammates do what I want them to do. • I am generally satisfied with my relationship with my teammates. • I think my relationship with my teammates is better than most peoples. • I sometimes wish I did not have a relationship with my teammates. (Reverse-coded) • My teammates meet my expectations. • I like my teammates. • There are problems between me and my teammates. (Reverse-coded)
4. Relationship with coach	• My relationship with my coach contributes to my personal growth. • There is a coach I know well. • I am satisfied with the coaching I receive from my coach. • There is a coach who gives me advice.
5. Satisfaction with home environment	• I am satisfied with my life at home.
6. Satisfaction with life	• In most ways my life is close to my ideal. • The conditions of my life are excellent. • I am satisfied with my life. • So far I have gotten the important things I want in life. • If I could live my life over, I would change almost nothing.
7. Satisfaction with time in daily life	• I enjoy my time in daily life.
8. Satisfaction with growth	• I am able to grow by being a member of this team.

All items were rated on a 5-point Likert scale (1 = strongly disagree, 2 = disagree, 3 = neither agree nor disagree, 4 = agree somewhat, 5 = strongly agree).

### 2.3 Procedure and data acquisition

The online psychological assessment was introduced to the target team in March 2024, and participation was recorded over the following year until March 2025. Although coaches encouraged athletes to complete the assessment, participation was conducted voluntarily based on the athletes’ own decisions.

Response data were exported by the system management department of Trois-re Co., Ltd., and then provided to the researchers. All personally identifiable information, including names, dates of birth, and email addresses, had been removed from the provided dataset. Additionally, repeated responses from the same athlete were linked using a unique ID.

### 2.4 Analysis perspectives and methods

To examine the effectiveness of the online psychological assessment, analyses were conducted from the following perspectives.

#### 2.4.1 Perspective 1: Monitoring participation

Balcombe and De Leo pointed out that, when conducting screening or follow-up assessments for athletes, the extent to which athletes continue to engage with the tool is a critical issue from both research and practical perspectives (2). However, reports on interventions using digital tools have often involved short-term use, with the period of use predetermined by researchers, and few studies have documented usage patterns over a long period when athletes use the tool voluntarily.

To investigate annual participation among the 81 athletes, the number of assessments completed by each athlete was tallied. The year was divided into four periods, aligned with the academic and competitive calendar: pre-season (March–June), first half of the season (July–September), second half of the season (October–December), and off-season (January–March). The number of assessments completed during each period was also recorded. From July to December was the competitive season, during which matches were concentrated mainly in regional league competitions and championships. An overview of the target team's annual schedule is presented in Supplementary Figure S2.

#### 2.4.2 Perspective 2: Overall trends and variability of psychological states

Tools for monitoring psychological states are often used for individual screening, but aggregating scores across items at the team level has been proposed as a means to capture the current state of the team. For example, Thornton and colleagues developed a monitoring system for training load and subjective fatigue, demonstrating that aggregated data can be effectively used by stakeholders to understand team status (14). Similarly, Saw and colleagues reported that simple self-reports from athletes can be used to assess overall team trends and inform decisions regarding training load adjustments and player rotation (15). To examine the overall trends of each measure in the online psychological assessment, mean scores and standard deviations were calculated for each measure, and descriptive statistics were performed for each of the four periods.

In addition, the variability of each measure was assessed. It is known that certain psychological measures fluctuate over short periods, while others remain relatively stable (16). Variability provides important information for determining the timing of interventions and evaluating their effects. Therefore, athletes who completed the assessment at least three times over the year were selected, and the absolute deviation of their individual scores was calculated. These absolute deviations were treated as variability scores, and the mean variability score was computed for each measure. Finally, to examine whether variability differed among the eight measures, the Friedman test was conducted.

#### 2.4.3 Perspective 3: Factors associated with key outcomes

Various studies have indicated associations between athletes’ psychological states and key outcomes in sports. For example, psychological factors affecting performance (17) and psychological traits related to motivation to participate and intent to continue participation (18) have been summarized in review articles. In particular, dropout from sports represents a significant concern for youth athletes (19). In this study, examining the extent to which measures of interpersonal relationships and overall life satisfaction explain motivation to continue participation allows for evaluation of the practical utility of the online psychological assessment tool.

To clarify the influence of satisfaction-related measures on motivation to continue participation, a mixed-effects linear regression model was conducted with motivation to continue participation as the dependent variable. Satisfaction measures were specified as fixed effects, and individual athletes were specified as random effects to account for repeated measures over time for each athlete. To identify measures most strongly associated with motivation, a stepwise variable selection procedure was applied: starting with a model including all seven explanatory variables, the variable with the smallest absolute standardized coefficient was sequentially removed. The optimal model was then determined based on marginal R^2^ (variance explained by fixed effects only), conditional *R*^2^ (variance explained including random effects), likelihood ratio tests, information criteria (AIC, BIC), and the significance of fixed-effect coefficients. In addition, variance inflation factors (VIFs) were calculated for each variable included as a fixed effect in the optimal model to assess multicollinearity.

### 2.5 Statistical analysis

For measures consisting of multiple items, reliability analyses were conducted to assess internal consistency. Item-total correlations for each item within a measure were calculated, and Cronbach's α coefficients were computed for the overall measure. Based on the criteria suggested by DeVellis, item-total correlations of 0.3 or higher and α coefficients of 0.6 or higher were confirmed (20). Additionally, the response time for each item—from its display on the screen to the participant's completion—was recorded.

All statistical analyses were performed using R version 4.4.0. Reliability analyses were conducted using the alpha function from the psych package (21), descriptive statistics using the describe function, and mixed-effects linear regression models using the lmer function from the lmerTest package (22). The significance level was set at 5%.

## 3 Results

### 3.1 Item analysis and response time

The results of the item analysis are presented in Supplementary Table S1. Item-total correlations exceeded 0.3 for all items, and Cronbach's *α* coefficients were above 0.6 for all measures. These results confirmed that internal consistency was adequately maintained for each measure. The mean response time per item was approximately 5–6 s, resulting in a total completion time of about 3 min. Considering the possibility that some screens were left displayed, leading to excessively long recorded times, the median response time was approximately 2–4 s per item. These findings suggest that, based on item difficulty and response time, youth athletes in junior high or high school were able to complete the assessment without undue burden.

### 3.2 Monitoring participation

Regarding participation in the online psychological assessment, a total of 337 assessments were completed over the one-year period, with a mean (SD) of 4.16 (2.67) assessments per athlete over the year. When categorized by the number of repeated assessments per athlete, 40 athletes (49.4%) completed 1–3 assessments, 24 athletes (29.6%) completed 4–6 assessments, 14 athletes (17.3%) completed 7–9 assessments, and 3 athletes (3.7%) completed 10 or more assessments. When examined by period, the number of assessments was 104 for pre-season (March–June), 102 for first half of the season (July–September), 70 for second half of the season (October–December), and 61 for off-season (January–March). Detailed participation data for each athlete can be found in Supplementary Table S2.

### 3.3 Overall trends and variability of psychological states

As shown in Table 2, descriptive statistics indicated that mean scores exceeded 4.0 for all measures except satisfaction with life. In particular, motivation to continue participation was high, with a mean (SD) of 4.74 (0.59), indicating that most youth athletes expressed a desire to continue participating in the academy. In contrast, standard deviations for relationship with coach and satisfaction with growth were observed to increase descriptively during the competitive season from July to December.

**Table 2:** Descriptive statistics of measures.

Measure	Statistics	Period				
		Overall	Pre-season	First half of the season	Second half of the season	Off-season
Motivation to continue participation	Mean	4.74	4.79	4.71	4.69	4.77
	SD	0.59	0.54	0.64	0.63	0.56
Relationship with family	Mean	4.47	4.47	4.49	4.59	4.30
	SD	0.64	0.69	0.58	0.59	0.67
Relationship with teammates	Mean	4.37	4.36	4.37	4.47	4.31
	SD	0.58	0.59	0.59	0.60	0.53
Relationship with coach	Mean	4.47	4.51	4.43	4.47	4.45
	SD	0.58	0.48	0.60	0.73	0.47
Satisfaction with home environment	Mean	4.53	4.62	4.53	4.53	4.38
	SD	0.81	0.75	0.84	0.85	0.80
Satisfaction with life	Mean	3.58	3.49	3.57	3.80	3.48
	SD	1.01	0.97	1.04	1.12	0.91
Satisfaction with time in daily life	Mean	4.37	4.35	4.46	4.49	4.11
	SD	0.82	0.91	0.77	0.78	0.78
Satisfaction with growth	Mean	4.67	4.74	4.64	4.60	4.70
	SD	0.63	0.52	0.67	0.71	0.64

The number of assessments was 337 for overall, 104 for pre-season (March–June), 102 for first half of the season (July–September), 70 for second half of the season (October–December), and 61 for off-season (January–March). The competitive season, divided in this study into the first half of the season and the second half of the season, during which mainly regional league and championship matches are held. An overview of the target team's annual schedule is presented in Supplementary Figure S2.

Table 3 presents the mean and distribution of within-individual variability scores for the eight measures. Results of the Friedman test revealed significant differences in variability scores among measures [*χ*^2^(7) = 28.73, *p* < 0.01]. Satisfaction with time in daily life (0.356) and satisfaction with life (0.354) showed higher variability scores, indicating greater within-individual fluctuations.

**Table 3:** Variability of measures.

Measure	Mean variability score	Distribution of variability scores						
		0.0–0.2	0.2–0.4	0.4–0.6	0.6–0.8	0.8–1.0	1.2–1.4	1.4–1.6
Satisfaction with time in daily life	0.356	17	7	20	0	0	0	1
Satisfaction with life	0.354	15	17	17	2	2	0	0
Satisfaction with home environment	0.269	29	5	11	1	6	1	0
Relationship with teammates	0.259	22	21	9	1	0	0	0
Relationship with coach	0.246	27	19	0	0	0	1	0
Satisfaction with growth	0.242	28	9	9	4	2	1	0
Motivation to continue participation	0.224	29	9	8	5	2	0	0
Relationship with family	0.203	32	13	6	2	0	0	0

The mean variability score was defined as the mean of within-person absolute deviations. A Friedman test revealed significant differences in variability scores across measures [*χ*^2^(7) = 28.73, *p* < 0.01].

### 3.4 Factors associated with motivation to continue participation

Table 4 presents the estimates from the mixed-effects linear regression models. Marginal R^2^ decreased from Model 5 onward. Likelihood ratio tests indicated significant differences for models from Model 5 onward, suggesting a decline in model fit as explanatory variables were reduced. Regarding information criteria, AIC was lowest for Model 2, while BIC was lowest for Model 5. All main effects of the explanatory variables included from Model 4 onward were significant. Considering these results comprehensively, Model 4, which included four explanatory variables, was selected as the optimal model. The model's R^2^ indicated that fixed effects alone explained 52.8% of the total variance, and including random effects explained 64.4% of the total variance. The influence of the four fixed-effect measures in Model 4 was highest for satisfaction with growth (standardized coefficient: 0.329), followed by relationship with coach (0.082), satisfaction with life (0.066), and satisfaction with time in daily life (0.057). VIFs were 1.483 for satisfaction with growth, 1.487 for relationship with coach, 1.475 for satisfaction with life, and 1.527 for satisfaction with time in daily life, indicating no variables exceeded the threshold of 5 that would suggest multicollinearity concerns.

**Table 4:** Linear mixed-effects model for motivation to continue participation.

Fit indices	Model 1	Model 2	Model 3	Model 4	Model 5	Model 6	Model 7
Marginal *R*^2^	0.536	0.536	0.530	0.528	0.517	0.495	0.454
Conditional *R*^2^	0.649	0.649	0.648	0.644	0.639	0.628	0.618
Likelihood ratio test (*p*-value)	–	0.987	0.135	0.131	0.040	0.003	*p* < 0.001
AIC	325.4	323.4	323.7	323.9	326.2	333.2	344.2
BIC	363.6	357.7	354.2	350.6	349.1	352.3	359.4
Standardized regression coefficient	Model 1	Model 2	Model 3	Model 4	Model 5	Model 6	Model 7
Satisfaction with growth	0.323**	0.323**	0.329**	0.329**	0.333**	0.375**	0.389**
Satisfaction with life	0.074*	0.074*	0.077*	0.066*	0.093**	0.101**	
Relationship with coach	0.069*	0.069*	0.084**	0.082**	0.087**		
Satisfaction with time in daily life	0.055	0.056	0.066*	0.057*			
Relationship with family	−0.046	−0.045	−0.041				
Relationship with teammates	0.044	0.044					
Satisfaction with home environment	0.001						

Marginal *R*^2^ indicates the proportion of variance explained by the fixed effects alone. Conditional *R*^2^ represents the proportion of variance explained by both fixed and random effects. Likelihood ratio tests compared consecutive models (e.g., Model 1 vs. Model 2, Model 2 vs. Model 3). For standardized regression coefficients, **indicates *p* < 0.01 and *indicates *p* < 0.05.

## 4 Discussion

When youth athletes in the soccer academy were allowed to participate voluntarily in the online psychological assessment, the average number of assessments completed over the one-year period was approximately four. Longitudinal studies examining key issues such as athlete burnout have often conducted multiple assessments at intervals of 1–3 months to capture changes in athletes’ psychological states (23). Based on this, it is considered desirable to conduct at least four assessments per year to monitor changes in psychological states and to identify the need for intervention. However, nearly half of the athletes (49.4%) completed three or fewer assessments, highlighting challenges in achieving continuous and regular use. When examined by period, the number of assessments decreased by 30%–40% in the latter half of the year, indicating a decline in athlete engagement over time. González-Barato and colleagues suggested that providing meaningful incentives to users is essential for sustaining engagement with digital tools (24). Although the tool used in this study provided immediate feedback on psychological scores, introducing additional incentives may be necessary. Furthermore, since athletes’ self-regulation abilities have been reported to influence continued use of digital tools (25), optimizing feedback according to individual psychological characteristics may also be effective. In addition to improvements in feedback and incentives, fatigue or boredom resulting from repeated assessments may also be a potential factor contributing to the decline in participation. The tool used in this study was designed to minimize respondent burden by simplifying the number of items, but further efforts to reduce the burden and maintain engagement may be necessary.

Descriptive statistics reflecting overall trends in psychological states are useful for understanding the current status of a team. Numerous studies have reported a positive association between team cohesion and performance in sports (26). Even focusing specifically on soccer, a large body of research has demonstrated that the stability and consistency of a team's psychological states are positively related to performance (27). In the present study, most of the scores for the eight measures exceeded 4.0, indicating that the majority of youth athletes responded positively to the items. Notably, no athletes withdrew from the team during the one-year study period, suggesting that overall motivation and satisfaction remained high.

On the other hand, during the competitive season, when maximal team performance is expected, standard deviations for relationship with coach and satisfaction with growth tended to increase. This indicates the emergence of variability in psychological states within the team. In soccer, differences in psychological states between regular and non-regular players (28), psychological impacts of injuries (29), and associations between performance decline and stress (30) have been reported. These factors may become more pronounced during the competitive season, potentially contributing to disparities in motivation and satisfaction among team members. Therefore, when interpreting assessment results, it is important not only to consider mean scores but also to account for the score variance within a team, enabling individualized support for athletes showing declines and strategies to promote team cohesion.

Regarding the variability of the measure scores, satisfaction with life and satisfaction with time in daily life were found to fluctuate considerably within individuals. Because these measures pertain to overall life outside team activities, they represent psychological states that are less visible to coaches. However, Stokes and colleagues emphasized the importance of supporting athletes’ non-athlete selves and showing interest in their overall well-being as a way to enhance their passion (31). To gain a deeper understanding of athletes, including psychological states that are highly variable and not easily observable during regular activities, regular administration of online psychological assessments is considered effective. Moreover, in considering intervention approaches based on psychological assessment results, it is important to distinguish factors that change over the short term from those that change over the long term. For example, in the case of competitive anxiety, state anxiety can be effectively addressed through immediate interventions that encourage positive reinterpretation, whereas trait anxiety requires long-term psychological support, including the acquisition of coping skills (32, 33). If short-term interventions are applied to psychological factors that fluctuate over the long term, unintended stress may arise for both athletes and coaches. Therefore, understanding the variability of psychological measures allows for the implementation of appropriate, tailored approaches.

In the field of sport, dropout among youth athletes has consistently been a critical issue during the developmental stages (34). Although various factors have been examined, previous studies have reported that, in addition to injuries and changes in family circumstances, psychological variables have a considerable influence on dropout (17). In the online psychological assessments used in this study, athletes’ surrounding relationships and overall life satisfaction explained more than 50% of the variance in motivation to continue participation. Specifically, high motivation to continue participation was clearly influenced by satisfaction with growth, relationship with coach, satisfaction with life, and satisfaction with time in daily life. Almagro and colleagues demonstrated that fulfilling the basic psychological need for competence, a fundamental need for athletes, enhances intrinsic motivation, which in turn promotes continued participation (35). Experiencing personal growth through team activities represents a key psychological factor that strengthens youth athletes’ motivation to continue participation. Concerning the relationship with the coach, a strong psychological connection has long been associated with higher adherence, improved performance, and greater well-being (36). The tool used in this study collected data based on athletes’ subjective evaluations. Accordingly, the relationship with the coach reflects whether athletes perceive dissatisfaction or concerns regarding their relationship with the team's coach. The findings suggest that preventing such negative psychological states is associated with the enhancement and maintenance of athletes’ motivation. Additionally, Han reported that athletes’ satisfaction with life may mitigate burnout via sport commitment and subjective vitality (37). For student youth athletes, time demands such as balancing sports and academics or commuting to venues can act as significant stressors, potentially leading to psychological and physical fatigue and dropout (38). The measures included in the online psychological assessments used in this study have been supported by previous research as effective for maintaining youth athletes’ motivation, and their effectiveness was also confirmed in the analyses of the present dataset.

Furthermore, based on the analyses of overall trends and within-individual variability reported above, satisfaction with growth and relationship with coach were measures that tended to fluctuate within the team during the competitive season. Therefore, appropriate care of these psychological states during the competitive season is likely to contribute to maintaining athletes’ motivation to continue participation. In addition, satisfaction with life and satisfaction with time in daily life were measures with high within-individual variability. Regular monitoring of psychological states outside of team activities may help prevent declines in motivation. Taken together, considering both the relationships among measures and their variability, continuous monitoring of psychological states can help implement strategies to prevent performance decline and dropout from the team.

Based on the findings of this study, the following practical recommendations are suggested for coaches to support athlete performance and well-being while utilizing an online psychological assessment tool:

- When using the assessment tool to monitor team psychological states, coaches should pay attention not only to central scores but also to the variability and changes in scores, in order to identify whether excessive divisions are occurring within the team or whether a small number of athletes are experiencing extreme deterioration in psychological states.
- Coaches should recognize that assessment tools are particularly useful for capturing athletes’ satisfaction in aspects of life outside team activities, which cannot be directly observed. They should also be aware that that athletes’ satisfaction in aspects of life outside team activities can fluctuate considerably over time.
- Information on athletes’ psychological states obtained through the assessment tool can provide meaningful insight into athletes’ motivation. In particular, the relationship between athletes and coaches, as well as athletes’ life satisfaction, can serve as valuable indicators. It is important to integrate observations of athletes’ actual behavior during activities, performance outcomes, and psychological information from the assessment tool in order to develop more effective strategies for support.

Finally, the limitations of this study and directions for future research are presented. This study focused on a single soccer academy consisting of youth athletes under the age of 18; therefore, caution is required when generalizing the findings. Patterns of digital tool utilization and psychological states may vary depending on factors such as team policies, coach characteristics, geographical conditions, and family environments. In this study, cases were reported from the perspective of using digital tools for online psychological assessment, and their applicability was examined. The usefulness of the findings was clarified based on overall trends and variability in psychological states, as well as the interrelationships among psychological factors. However, no specific analyses were conducted that directly examined how athletes and coaches actually used the assessment results, or how the introduction of digital tools influenced team practices and dynamics. Based on these considerations, two directions for future research can be proposed. First, to examine psychological assessment tools while accounting for differences in team characteristics, it is necessary to accumulate case studies across a wide range of teams. Second, longitudinal research that follows the same team over an extended period should be conducted to clearly identify the effects of assessment tool usage on athletes’ performance, sport participation continuity, and well-being. By collecting and analyzing team-based implementation cases more broadly and continuously, it will be possible to comprehensively evaluate the effectiveness of psychological assessment tools.

## Data Availability

The raw data supporting the conclusions of this article will be made available by the authors, without undue reservation.

## References

[1] Breslin G Shannon S Haughey T Donnelly P Leavey G. A systematic review of interventions to increase awareness of mental health and well-being in athletes, coaches and officials. Syst Rev. (2017) 6:177. 10.1186/s13643-017-0568-6 28859666 PMC5579872

[2] Balcombe L De Leo D. Psychological screening and tracking of athletes and digital mental health solutions in a hybrid model of care: mini review. JMIR Form Res. (2020) 4(12):e22755. 10.2196/22755 33271497 PMC7746225

[3] Collins R Yang R D’Souza BN Gore A Bay C Erondu I Mobile app use and the mental health of elite athletes. Sports Psychiatry. (2025) 4(3):131–38. 10.1024/2674-0052/a000104

[4] Kittler C Stenzel L Jekauc D Stoll O. Implementation of an app-based blended mindfulness intervention in a bundesliga youth academy targeting goalkeepers: a case study. Case Studies Sport Exerc Psychol. (2021) 5(1):95–105. 10.1123/cssep.2021-0006

[5] Lee YH Chiu W Hwang J Noh S. Mobile-based mindfulness meditation intervention’s impact on mental health among young male judo athletes in South Korea: a quasi-experimental study. Sci Rep. (2024) 14(1):12691. 10.1038/s41598-024-63637-0 38830986 PMC11148055

[6] Bonetti R Rod B Hauw D. Quality of mobile apps for psychological skills training in sport: a MARS-based study. Comput Hum Behav Rep. (2025) 18:100635. 10.1016/j.chbr.2025.100635

[7] Anderson T Adams WM Bartley JD Brutus AL Donaldson AT Finnoff JT. Analysis of the sport mental health assessment tool 1 (SMHAT-1) in team USA athletes. Br J Sports Med. (2023) 57(18):1187–94. 10.1136/bjsports-2022-106495 37369554 PMC10579191

[8] Shannon S Shevlin M Brick N Donnelly P Horgan P Breslin G. Psychometric analysis of the international Olympic committee’s sport mental health assessment triage tool among non-elite amateur adult athletes. Int J Sport & Exerc Psychol. (2024) 23(4):553–74. 10.1080/1612197X.2024.2335226

[9] Waleriańczyk W Krzywański J Gorgol J Konopka K Krysztofiak H Kuśmierczyk A Diagnostic effectiveness of the sport mental health assessment tool 1 supplemented with a brief clinical intake interview in a cohort of Polish elite Olympic athletes. Br J Sports Med. (2024) 59(1):56–63. 10.1136/bjsports-2024-108919 39443072

[10] Donohue B Scott J Goodwin G Barchard KA Bohall G Allen DN. Initial examination of the mental health disorders: screening instrument for athletes. Front. Psychol. (2023) 14:1029229. 10.3389/fpsyg.2023.1029229 37599751 PMC10436329

[11] Keegan R Harwood C Spray CM Lavallee D. A qualitative investigation exploring the motivational climate in early career sports participants: coach, parent and peer influences on sport motivation. Psychol Sport Exerc. (2009) 10(3):361–72. 10.1016/j.psychsport.2008.12.003

[12] Valkenburg PM Sumter SR Peter J. Gender differences in online and offline self-disclosure in pre-adolescence and adolescence. Br J Dev Psychol. (2011) 29(2):253–69. 10.1348/204483510X501994 21199497

[13] Sano T Matsuoka R Momota M Hamano Y. Actual Status of the relationship between motivation for activities and satisfaction level as seen from the implementation of online psychological tests in sports teams: a case study of sports teams targeting junior high school children. Sports Industry Stud. (2024) 34(3):225–38. 10.5997/sposun.34.3_225

[14] Thornton HR Delaney JA Duthie GM Dascombe BJ. Developing athlete monitoring systems in team sports: data analysis and visualization. Int J Sports Physiol Perform. (2019) 14(6):698–705. 10.1123/ijspp.2018-0169 30676144

[15] Saw AE Main LC Gastin PB. Monitoring the athlete training response: subjective self-reported measures trump commonly used objective measures: a systematic review. Br J Sports Med. (2016) 50(5):281–91. 10.1136/bjsports-2015-094758 26423706 PMC4789708

[16] Geiser C Götz T Preckel F Freund PA. States and traits: theories, models, and assessment. Eur J Psychol Assess. (2017) 33(4):219–23. 10.1027/1015-5759/a000413

[17] Lochbaum M Sisneros C Cooper S Terry PC. Pre-event self-efficacy and sports performance: a systematic review with meta-analysis. Sports. (2023) 11(11):222. 10.3390/sports11110222 37999439 PMC10675036

[18] Zhang M Wang Y Zhang X Zhang T Zhang J Liu Y. Predictors of persistent participation in youth sport: a systematic review and meta-analysis. Front Psychol. (2022) 13:871936. 10.3389/fpsyg.2022.871936 35712153 PMC9196305

[19] Crane J Temple V. A systematic review of dropout from organized sport among children and youth. Eur Phys Educ Rev. (2015) 21(1):114–31. 10.1177/1356336X14555294

[20] DeVellis RF Thorpe CT. Scale Development: Theory and Applications. 5th ed Thousand Oaks, CA: SAGE Publications (2021).

[21] Revelle W. Psych: Procedures for Psychological, Psychometric, and Personality Research (R Package). Evanston, IL: Northwestern University (2018).

[22] Kuznetsova A Brockhoff PB Christensen RHB. Lmertest package: tests in linear mixed effects models. J Stat Softw. (2017) 82(13):1–26. 10.18637/jss.v082.i13

[23] Dišlere BE Mārtinsone K Koļesņikova J. A scoping review of longitudinal studies of athlete burnout. Front Psychol. (2025) 16:1502174. 10.3389/fpsyg.2025.1502174 40028645 PMC11868109

[24] González-Barato LJ Rubio VJ Hernández JM Sánchez-Iglesias I. PSIXPORT: mobile app for ecological momentary assessment of psychological dimensions in sport injury. Front Sports Act Living. (2021) 12:697293. 10.3389/fpsyg.2021.697293 PMC8353149 34385962

[25] Jakowski S. Self-tracking via smartphone app: potential tool for athletes’ recovery self-management? Ger J Exerc Sport Res. (2022) 52:253–61. 10.1007/s12662-022-00812-3 40477594 PMC9053116

[26] Carron AV Colman MM Wheeler J Stevens D. Cohesion and performance in sport: a meta-analysis. J Sport Exerc Psychol. (2002) 24(2):168–88. 10.1123/jsep.24.2.168

[27] Alves M. Collective efficacy in soccer teams: a systematic review. Psychol Res Behav Manag. (2021) 14:1–15. 10.2147/PRBM.S314358 34173889 PMC8236009

[28] Alix-Sy D Le Scanff C Filaire E. Psychophysiological responses in the pre-competition period in elite soccer players. J Sports Sci Med. (2008) 7(4):446–54. 10.52082/jssm.2008.446 24149949 PMC3761908

[29] Baize D d’Arripe-Longueville F Piponnier E Scoffier-Meriaux S. Psychological risk factors for a first hamstring strain injury in soccer: a qualitative study. Front Sports Act Living. (2024) 6:1377045. 10.3389/fspor.2024.1377045 38947866 PMC11211564

[30] Mallas GR Heilbrun K. The relationship between perceived stress, personality, and performance in professional soccer players. J Appl Sport Psychol. (2019) 31(4):421–36. 10.1080/10413200.2018.1554395

[31] Stokes AT Cain E Martin EM. “I can’t imagine not being an athlete”: a retrospective qualitative analysis of the factors that influence sport passion development in collegiate athletes. J Adv Sport Psychol Res. (2024) 4(2):4–19. 10.55743/000026

[32] Li H Yang Q Wang B. Effects of psychological interventions on anxiety in athletes: a meta-analysis based on controlled trials. Front Psychol. (2025) 16:1621635. 10.3389/fpsyg.2025.1621635 40851578 PMC12368976

[33] Horikawa M Yagi A. The relationships among trait anxiety, state anxiety and the goal performance of penalty shoot-out by university soccer players. PLoS One. (2012) 7(4):e35727. 10.1371/journal.pone.0035727 22539998 PMC3335041

[34] Soares ALA Carvalho HM. Burnout and dropout associated with talent development in youth sports. Front Sports Act Living. (2023) 5:1190453. 10.3389/fspor.2023.1190453 37334016 PMC10272455

[35] Almagro BJ Sáenz-López P Fierro-Suero S Conde C. Perceived performance, intrinsic motivation and adherence in athletes. Int J Environ Res Public Health. (2020) 17(24):9441. 10.3390/ijerph17249441 33339278 PMC7767293

[36] Mageau GA Vallerand RJ. The coach–athlete relationship: a motivational model. J Sports Sci. (2003) 21(11):883–904. 10.1080/0264041031000140374 14626368

[37] Han MT. The effect of sport life satisfaction on athlete burnout: the serial mediating role of sport commitment and subjective vitality. J Sport Sci Res. (2025) 10(3):406–28. 10.56423/jssr.2025.10.3.406

[38] De Maio M Montenegro S Papale O Serafini S Prestanti I Izzicupo P A scoping review of student Athletes’ perspectives on dual career policies, provisions and challenges. Front Sports Act Living. (2025) 7:1566208. 10.3389/fspor.2025.1566208 40529403 PMC12170630

