# Supplementary material for: Implementation and utility of an online psychological assessment tool in youth soccer players: a one-year longitudinal study
Source: Front Sports Act Living. 2026 Jan 26;8:1733902. doi: 10.3389/fspor.2026.1733902 (PMC12883758; doi:10.3389/fspor.2026.1733902)
Supplement: Supplementary file 1 [file Table1.docx]

Supplementary Table 1　Item analysis and response time.

| Measure | Item | Item–total correlation | Response time (s) | |
| --- | --- | --- | --- | --- |
|  |  |  | Mean | Median |
| Motivation to continue participation | I want to continue participating in this team’s activities. | - | 5.67 | 3.33 |
| Relationship with | My family does what I want them to do. | 0.73 | 4.81 | 3.60 |
| family | I am generally satisfied with my relationship with my family. | 0.82 | 5.37 | 2.79 |
| Cronbach’s α=0.872 | I think my relationship with my family is better than most people’s. | 0.79 | 5.19 | 3.45 |
|  | I sometimes wish I did not have a relationship with my family. ^†^ | 0.65 | 6.73 | 3.83 |
|  | My family meets my expectations. | 0.81 | 5.03 | 2.99 |
|  | I like my family. | 0.81 | 3.93 | 2.30 |
|  | There are problems between me and my family.^†^ | 0.73 | 5.51 | 3.39 |
| Relationship with | My teammates do what I want them to do. | 0.72 | 8.75 | 3.75 |
| teammates | I am generally satisfied with my relationship with my teammates. | 0.75 | 4.61 | 2.59 |
| Cronbach’s α＝0.784 | I think my relationship with my teammates is better than most people’s. | 0.69 | 6.08 | 3.57 |
|  | I sometimes wish I did not have a relationship with my teammates.^†^ | 0.56 | 6.85 | 4.46 |
|  | My teammates meet my expectations. | 0.70 | 6.18 | 3.94 |
|  | I like my teammates. | 0.78 | 5.02 | 2.19 |
|  | There are problems between me and my teammates. ^†^ | 0.60 | 6.34 | 4.08 |
| Relationship with | My relationship with my coach contributes to my personal growth. | 0.78 | 4.94 | 3.38 |
| coach | There is a coach I know well. | 0.74 | 4.57 | 3.26 |
| Cronbach’s α＝0.645 | I am satisfied with the coaching I receive from my coach. | 0.79 | 3.26 | 2.00 |
|  | There is a coach who gives me advice. | 0.69 | 2.97 | 2.10 |
| Satisfaction with home environment | I am satisfied with my life at home. | - | 4.56 | 2.93 |
| Satisfaction with life | In most ways my life is close to my ideal. | 0.86 | 4.69 | 3.59 |
| Cronbach’s α＝0.867 | The conditions of my life are excellent. | 0.86 | 3.99 | 2.25 |
|  | I am satisfied with my life. | 0.81 | 4.29 | 2.89 |
|  | So far I have gotten the important things I want in life. | 0.75 | 5.02 | 3.40 |
|  | If I could live my life over, I would change almost nothing. | 0.79 | 5.44 | 3.48 |
| Satisfaction with time in daily life | I enjoy my time in daily life. | - | 5.74 | 3.85 |
| Satisfaction with growth | I am able to grow by being a member of this team. | - | 2.94 | 2.07 |

^†^Reverse-coded items. For multi-item measures, item–total correlations and Cronbach’s alpha coefficients were calculated.
