# Supplementary material for: Implementation and utility of an online psychological assessment tool in youth soccer players: a one-year longitudinal study
Source: Front Sports Act Living. 2026 Jan 26;8:1733902. doi: 10.3389/fspor.2026.1733902 (PMC12883758; doi:10.3389/fspor.2026.1733902)
Supplement: Supplementary file 3 [file Datasheet1.pdf]

Online psychological assessment procedure

1. Access the assessment QR code via a computer, smartphone, or tablet.

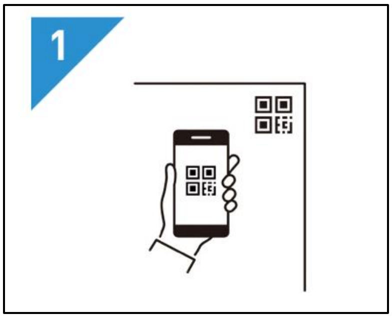

2. Enter the required information, check the consent screen, and start the assessment.

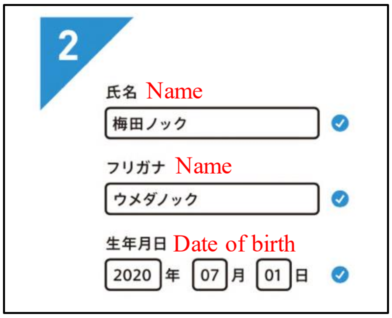

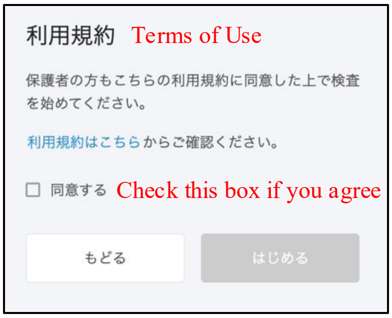

3. Respond to the items presented in a questionnaire format.

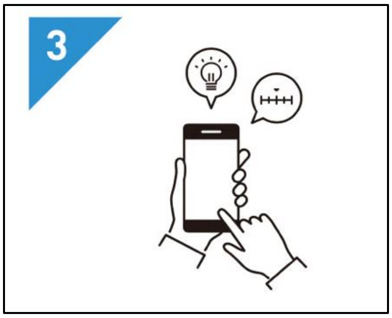

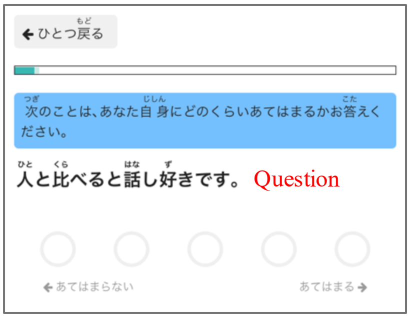

4. Upon completion, the results are immediately provided as feedback to the respondent.
5. Coaches with administrator privileges can view all athletes' results within the team.

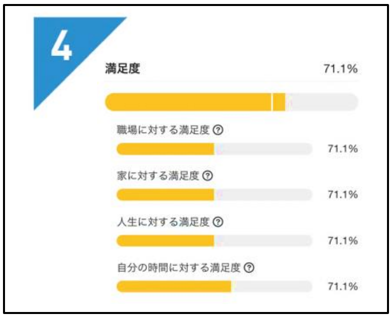

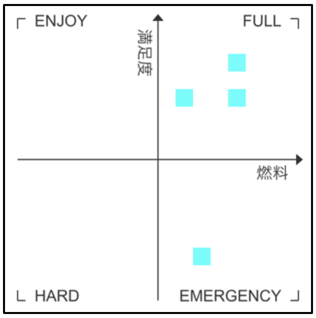

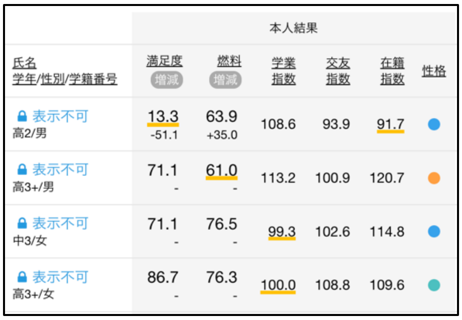

氏名 学年/性別/学籍番号	本人結果 満足度 増減	燃料 増減	学業指数	交友指数	在籍指数	性格
表示不可 高2/男	13.3 -51.1	63.9 +35.0	108.6	93.9	91.7	●
表示不可 高3+/男	71.1 -	61.0 -	113.2	100.9	120.7	●
表示不可 中3/女	71.1 -	76.5 -	99.3	102.6	114.8	●
表示不可 高3+/女	86.7 -	76.3 -	100.0	108.8	109.6	●
