# Supplementary figures and images for: Implementation and utility of an online psychological assessment tool in youth soccer players: a one-year longitudinal study
Source: Front Sports Act Living. 2026 Jan 26;8:1733902. doi: 10.3389/fspor.2026.1733902 (PMC12883758; doi:10.3389/fspor.2026.1733902)

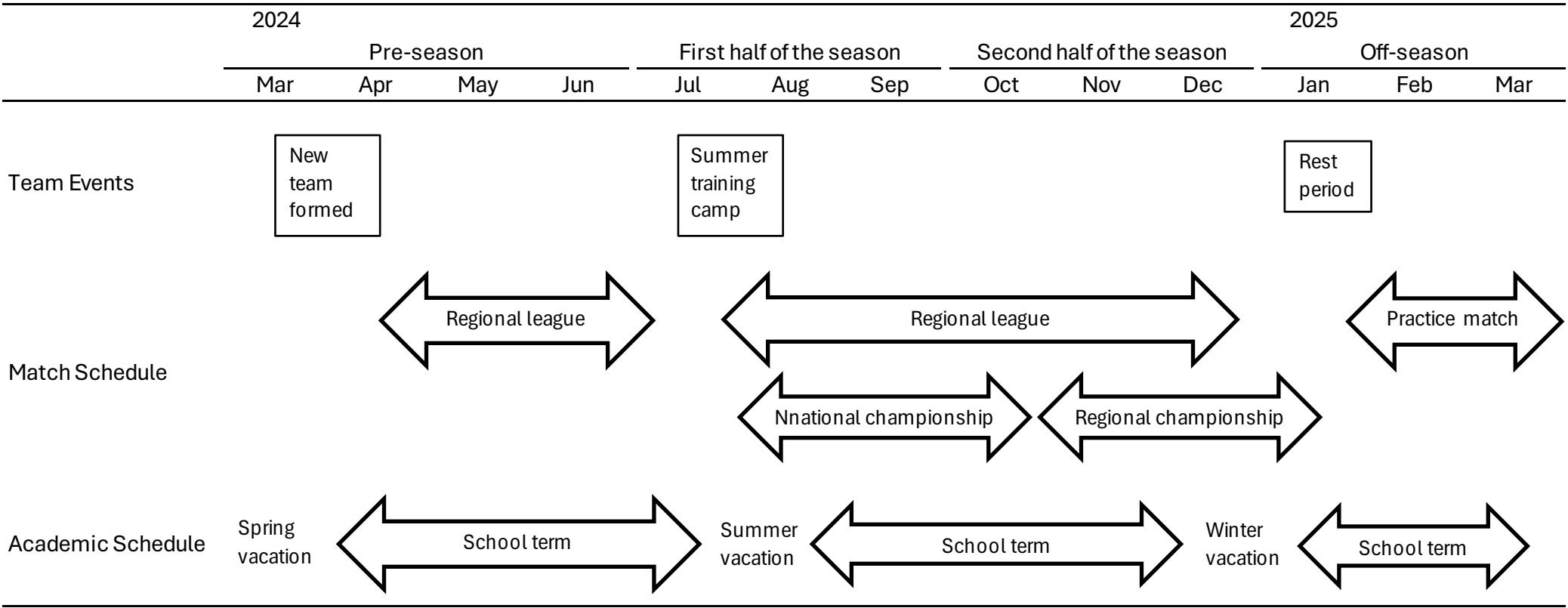

Supplement: Supplementary file 4 [file Datasheet2.pdf]
